# Biodiversity of *Trichoderma* from grassland and forest ecosystems in Northern Xinjiang, China

**DOI:** 10.1007/s13205-020-02301-6

**Published:** 2020-07-30

**Authors:** Jing Ma, Estifanos Tsegaye, Mei Li, Beilei Wu, Xiliang Jiang

**Affiliations:** Institute of Plant Protection, Chinese Academy of Agricultural Sciences, No. 2, West Yuanmingyuan Rd., Haidian District, Beijing, 100193 China

**Keywords:** *Trichoderma* spp., Biodiversity, Northern Xinjiang, Grassland and forest, Altitude

## Abstract

*Trichoderma* spp., a cosmopolitan fungal genus, has remarkable economic value in industry and agriculture. The resources of *Trichoderma* spp. in the grassland and forest ecosystems of northern Xinjiang were explored in this study. A total of 634 soil samples was collected, and 312 strains assigned to 23 species of *Trichoderma* spp. were identified. *T. harzianum* was the dominant species with 28.2% from all isolates. The principal components analysis indicated that ecosystem was the most dominant impact factor among longitude, latitude, altitude and ecosystems for the species diversities of *Trichoderma* spp. with the decreasing trend from the north to the south of northern Xinjiang (e.g., from Altay, followed by Yili, Changji, Bayingolin and finally Urumqi). Overall, *Trichoderma* spp. were more frequently encountered in forest ecosystems (coniferous forest and coniferous and broadleaf mixed forest) than in grassland ecosystems (desert steppe and temperate steppe). Frequency of *Trichoderma* spp. was significantly decreased along with increased altitude and only a few strains were isolated from altitudes above 3000 m. The results provided essential information on *Trichoderma* occurrence and distribution, which should benefit the application of *Trichoderma* in agriculture.

**Electronic supplementary material:**

The online version of this article (10.1007/s13205-020-02301-6) contains supplementary material, which is available to authorized users.

## Introduction

As a cosmopolitan fungal genus, *Trichoderma* (*Ascomycetes*, *Hypocreales*) spp. are found in different environments, such as soil, above-ground plants, fungal material, decaying wood, sediment, and various other substances (Cummings et al. 2016; Harman 2000; Jaklitsch 2009; Jaklitsch and Voglmayr 2015; Kubicek et al. 2008). Many studies have been published about promising applications of *Trichoderma* spp. in industry and agriculture due to their ability to produce enzymes and antibiotics, promote plant growth, induce plant resistance (Gajera et al. 2015; Mukherjee et al. 2013; Ortega-García et al. 2015; Vitti et al. 2016), and enhance the efficiency of nutrient use (Kashyap et al. 2017). In particular, *Trichoderma* strains are significant biocontrol agents against plant fungal pathogens(Bae et al. 2016; El-Hassan et al. 2013; Harman et al. 2004; Zhang et al. 2015a, b).

Recently, biodiversity of *Trichoderma* has been extensively investigated in different countries and regions, including Russia, Siberia, the Himalayas (Kullnig et al. 2000), a mid-European area (Wuczkowski et al. 2003), Egypt (Gherbawy et al. 2004), Tenerife (Zachow et al. 2009a, b), Colombia (Hoyos-Carvajal et al. 2009), Iran (Naeimi et al. 2011), South-East Asian (Kubicek et al. 2003), Manipur (Kamala et al. 2015), Southern Europe and Macaronesia (Jaklitsch and Voglmayr 2015), Malaysian Borneo (Cummings et al. 2016), and New Zealand (Braithwaite et al. 2017). These studies focused on the species diversity, phylogeny, geographic distribution, and habitat preference of *Trichoderma* as well as the effect of collection season and crop type on the distribution of *Trichoderma* spp. (Chaverri and Samuels 2013; Jiang et al. 2016; Kubicek et al. 2008). In China, species diversity and the distribution of *Trichoderma* have been investigated nationwide since the 1990s (Wen et al. 1993). Zhang et al. identified northern China as a potential center of origin of a unique haplotype of *T. harzianum* (Zhang et al. 2005a, b). Sun et al. further identified 23 *Trichoderma* spp. with significant ecological, biochemical, and genetic diversity in north, southwest, southeast, and middle China,and found that *T. harzianum* was the most widely distributed species in China, and the highest biodiversity of *Trichoderma* populations occurred in southwest China (Sun et al. 2012). In 2016, Jiang et al. surveyed *Trichoderma* biodiversity of 17 species in agricultural fields in four provinces of eastern China (Jiang et al. 2016).

The Xinjiang Uygur Autonomous Region in Northwestern China (73°E–96°E, 34°N–48°N) covers an area of 1.66 million square kilometers, and has rich natural resources because of its unique geographical and ecological environment, complex and diverse landforms and soils. Tianshan and Altai Mountains in northern Xinjiang are also covered with luxuriantly green primary forests and vast grasslands. The complex terrain and diverse ecological environment in this region provide capacity for *Trichoderm*a spp. biodiversity. However, no system research have been conducted on the diversity of *Trichoderma* spp. In this study, we focused on the recovery of *Trichoderma* diversities in grassland (desert steppe and temperate steppe) and forest (coniferous forest and coniferous and broadleaf mixed forest) ecosystems of northern Xinjiang based on altitudes, longitude, latitude and ecosystems.

## Methods

### Study regions and sample collection

A total of 634 soil samples were collected in July 2014, 2015, and 2016 from grassland (desert steppe and temperate steppe) and forest (coniferous forest and coniferous and broadleaf mixed forest) ecosystems in Xinjiang Uygur Autonomous Region. This region consists of Urumqi Nanshan (hereinafter referred to as Urumqi), Changji Tianchi (Changji), Ili Kazak Autonomous Prefecture (Yili), Altay Prefecture (Altay), and Mongolian Autonomous Prefecture of Bayingolin (Bayingolin). Each sample contained about 200 g of mixed soil from all five locations covering about 400 m^2^, a depth of approximately 5−20 cm. The longitude, latitude, and altitude of the collection locations, vegetation families, and geographical coordinates were recorded and are shown in Table S1. Samples were placed into sterile polyethylene bags, transported to the laboratory, and stored at 4 °C.

### Isolation and storage of *Trichoderma* strains

PDAm(Vargas Gil et al. 2009) and Rose Bengal Agar were used as selective media and *Trichoderma* strains were then isolated using the soil dilution plating method; colonial morphology was observed after 5–10 days. *Trichoderma* and non-*Trichoderma* colonies were observed. Putative *Trichoderma* colonies were purified by two rounds of subculture on potato-dextrose agar (PDA). All isolates described in this study were stored in liquid storage medium containing glycerol (final concentration 20%).

### Identification of *Trichoderma* spp.

Species were identified with a combination of morphological analysis and molecular methods. The morphological characteristics were based on the key by Gams and Bissett(Gams 1998). Colony characteristics were examined in cultures grown on PDA, CMD, and SNA, after 10 days of incubation at 25 °C. Microscopic observations were performed in cultures grown on PDA. As recommended by Gazis (Gazis et al. 2011), molecular identification was first done based on sequences of internal transcribed spacer regions 1 and 2 (ITS1 and ITS2) of the rRNA gene cluster. In case of failure in unambiguous species identification with ITS1 and ITS2, we also sequenced a fragment of the translation elongation factor 1-alpha (*tef1*-*α*) gene and the RNA polymerase II subunit B (*rpb2*) gene for further identification. Mycelia for DNA extraction were obtained on PDA through 5–7 days of incubation at 28 °C in a mildew incubator. Total DNA was extracted using the Fungal DNA Kit (Aidlab Biotechnologies Co., Ltd).

The ITS region of the rDNA was amplified using primers ITS4 and ITS5(White et al. 1990). A fragment of the *tef1* gene was amplified using the primers TEF1-728F(Druzhinina et al. 2004) and TEF1LLErev(Jaklitsch et al. 2005). A fragment of *rpb2* was amplified using the primer pair RPB2-250 (forward) and RPB2-1150 (reverse). Amplifications were performed in either a PTC-200 or PTC-100 thermocycler (MJ Research, USA) under the following conditions: initial denaturation 3 min at 94 °C, 35 cycles of 1 min at 94 °C, 1 min at 49 °C (for the ITS region), or 55 °C (for the *tef1*-*α* and *rpb2* fragment), 1 min at 72 °C, with a final extension of 10 min at 72 °C.

### Sequence and phylogenetic analysis

ITS rDNA sequences of all isolates were submitted to the oligonucleotide barcode program TrichOKEY(Druzhinina et al. 2005), *tef1* and *rpb2* sequences were submitted to TrichoBLAST(Kopchinskiy et al. 2005) and blasted in NCBI (https://www.ncbi.nlm.nih.gov/). ITS rDNA sequences of all *Trichoderma* strains were used to obtain haplotypes in Dna.sp ver. 5.1(Librado and Rozas 2009). Sequences of known species including type strains and outgroup were downloaded from the NCBI database. Type haplotypes and known species were aligned using Clustal W(Larkin et al. 2007), and then rechecked and adjusted manually as necessary using MEGA6.0 software(Tamura et al. 2013). Phylogenetic relationships were reconstructed with MEGA6.0 using the maximum likelihood approach (Kimura 2-parameter model and gamma distributed, with complete deletion in gaps/missing data treatment). All reconstructions were tested with 1000 bootstrap replicates. All sequences were deposited in GenBank with the accession numbers given in Supplemental Table 1.

### Diversity analysis

The degree of dominance index (Y) was used to quantitatively describe the adaptation of *Trichoderma* to all fungi in soil. The dominance values were calculated using the following formula:

$$ {\text{Y}} = \frac{{n_{i} }}{N} \times f_{i} , $$

where ‘N’ is the total count of fungal strains, ‘n_*i*_’ is the count of genus (species) *i*, and ‘*f*_*i*_’ is the frequency with which genus (species) *i* appears in the samples. The genus or species *i* is dominant when Y > 0.02.

Simpson biodiversity index (Dr)(Simpson 1949), Shannon–Weiner biodiversity index (H)(Shannon 1948), Pielou species evenness index (J)(Pielou 1966) and Margalef’s abundance index (E)(Margalef 1958), were used to quantitatively describe the diversity of *Trichoderma* species in different environments and regions. Margalef’s abundance index was used to represent richness, Simpson index was applied as a measure of the probability of diversity, the Shannon–Wiener index was followed to measure community diversity, and the Pielou index was used to measure evenness of the community. The calculation formulas of the biological diversity indexes are as follows(Gomes et al. 2018; Geml et al. 2014; Shi et al. 2014):

$$ {\text{Dr}} = \frac{1}{{\varSigma_{{{\text{i}} - 1}}^{s} P_{i}^{2} }},\;{\text{Pi}}^{2} = \frac{{n_{i} \left( {n_{i} - 1} \right)}}{{N\left( {N - 1} \right)}} $$

$$ {\text{H}} = - \sum_{i = 1}^{N }  P_{i} \ln P_{i} ,\;P_{i} = \frac{{n_{i} }}{N} $$

$$ {\text{J}} = \frac{H}{{H_{max} }},\;H_{max} = \ln S $$

$$ E = \frac{S - 1}{ln\;N}, $$

where ‘S’ is the number of *Trichoderma* species, ‘N’ is the sum of all *Trichoderma* species strains, ‘Pi’ is relative quantity of *Trichoderma* species ‘i’, and ‘n_i_’ is the number of strains of *Trichoderma* species ‘i’. All statistical analyses were performed using Microsoft Excel 2010.

### Principal components analysis

To determine the dominant factor among altitudes, longitudes, latitudes and ecosystems for the distribution of *Trichoderm*a spp., a principal components analysis (PCA) was conducted in R package vegan (Garrido-Benavent et al. 2020).

### Data availability

All data generated or analyzed during this study are included in this published article (and its Supplementary Information files).

## Results

### *Trichoderma* isolation and species identification

We obtained 2,859 *Trichoderma* isolates from samples collected from five regions of northern Xinjiang in three consecutive years from 2014 to 2016. Due to the large number of isolates, the fungi were initially grouped according to morphological characteristics (type of mycelium, colony color, presence of spores) and then representative isolates with the same morphological characteristics from the soil sample were selected for subsequent analysis, resulting in 312 *Trichoderma* isolates. With the combination of morphological analysis and molecular methods based on the ITS, *tef1*-*α* and *rpb2* genes, we identified 23 species: *T. harzianum* (88 strains), *T. paraviridescens* (46), *T. longibrachiatum* (26), *T. polysporum* (24), *T. asperellum* (20), *T. afroharzianum* (20), *T. oblongisporum* (17), *T. citrinoviride* (17), *T. rossicum* (14), *T. viridescens* (7), *T. saturnisporum* (6), *T. gamsii* (5), *T. semiorbis* (4), *T. pleurotum* (3), *T. koningii* (3), *T. atroviride* (3), *T. ghanense* (2), *T. brevicompactum* (2), *T. piluliferum* (1), *T. hamatum* (1), *T. pararogersonii* (1), *T. fertile* (1), and *T. caerulescens* (1) (Supplemental Table 1). Of these species, *T. pararogersonii* was identified as a new species record in China.

### Phylogenetic analysis

**Fig. 1:**
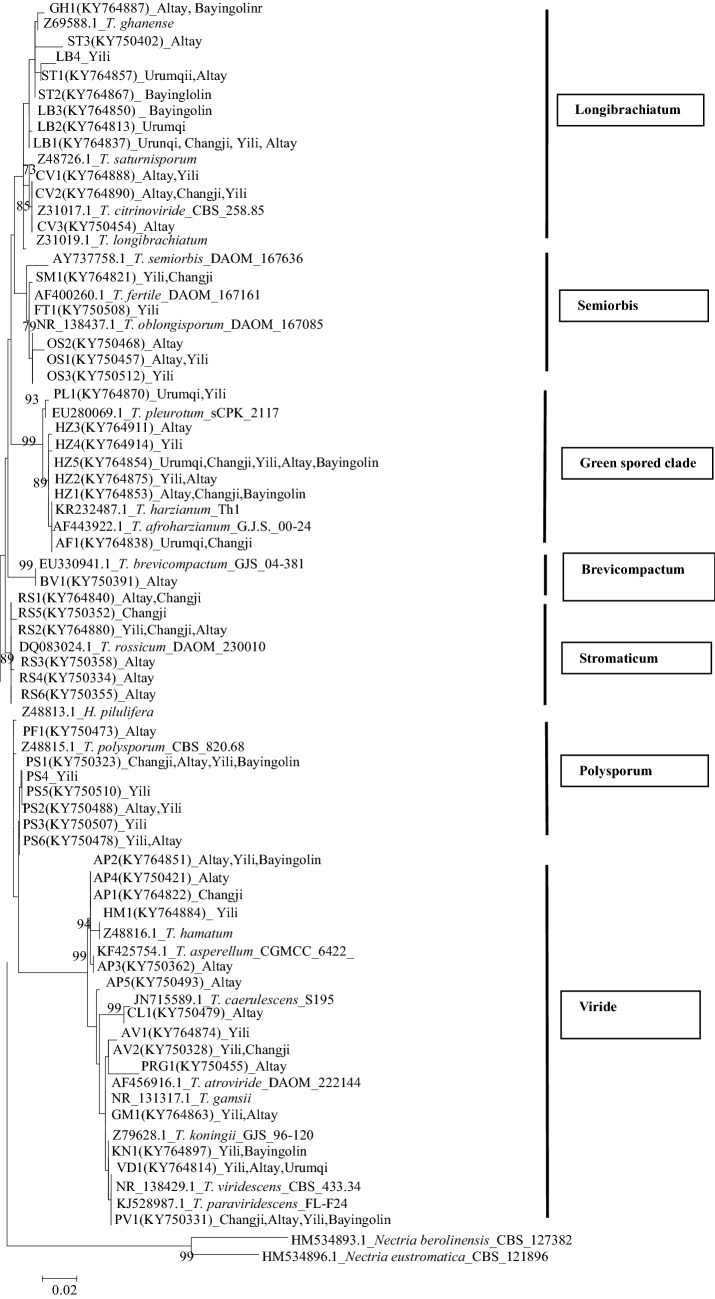
Phylogenetic tree inferred from ITS rDNA sequences using maximum likelihood with outgroup of *Nectria* spp. under 1000 bootstrap replicates analyzed by MEGA 7 Version

To infer a phylogenetic tree, we first calculated haplotypes from ITS1 and ITS2 sequences of the 312 strains. Finally, 51 haplotypes (Supplemental Table 1) were subjected to maximum likelihood analysis; *Nectria eustromatica* and *N. berolinensis* were used as outgroup taxa to root the tree. The phylogenetic tree is shown in Fig. 1. The 51 haplotypes belonging to the 23 *Trichoderma* species were placed in seven groups. The phylogenetic structure was consistent with previously established sections and clades in most cases(Druzhinina et al. 2006; Druzhinina et al. 2012).

The first group included the Viride clade with *T. asperellum*, *T. hamatum*, *T. caerulescens*, *T. atroviride*, *T. gamsii*, *T. koningii*, *T. viridescens*, and *T. paraviridescens*. Within this clade, *T. asperellum* and *T. hamatum* (except AP5) formed a separate branch named the Hamatum Clade as described previously(Druzhinina et al. 2006). However, many of the species on the first branch, eventually identified from analysis of *tef1*-*α* sequences and phenetic data, which were not differentiated by the phylogenetic analysis of their ITS sequences: (a) *T. gamsii* and *T. koningii*; (b) *T. paraviridescens* and *T. viridescens*; (c) *T. caerulescens*; and (d) *T. pararogersonii*. The second group, lacking bootstrap support, comprises two species in clade Polysporum- *T. polysporum* and *T. piluliferum*. The six haplotypes of *T. rossicum* constituted the third group with a bootstrap value of 89. The fourth group is clade Brevicompactum, which only includes one species (*T. brevicompactum*) with strong bootstrap support (99%). The fifth group is the Green spored clade, including *T. harzianum*, *T. afroharzianum* and *T. pleurotum*; this clade had 99% bootstrap support. Within this clade, the six haplotypes of *T. harzianum* formed a moderately well-supported (89%) clade. The sixth group (Semiorbis), which lacked significant bootstrap support, includes *T. semiorbis*, *T. fertile*, and *T. oblongisporum*. Within Semiorbis, three haplotypes of *T. oblongisporum* formed a separate clade with 79% bootstrap support. The last group (clade Longibrachiatum) includes *T. longibrachiatum*, *T. ghanense*, *T. saturnisporum*, and *T. citrinoviride*, and had low support (73%).

### Species diversity, evenness, and abundance

**Fig. 2:**
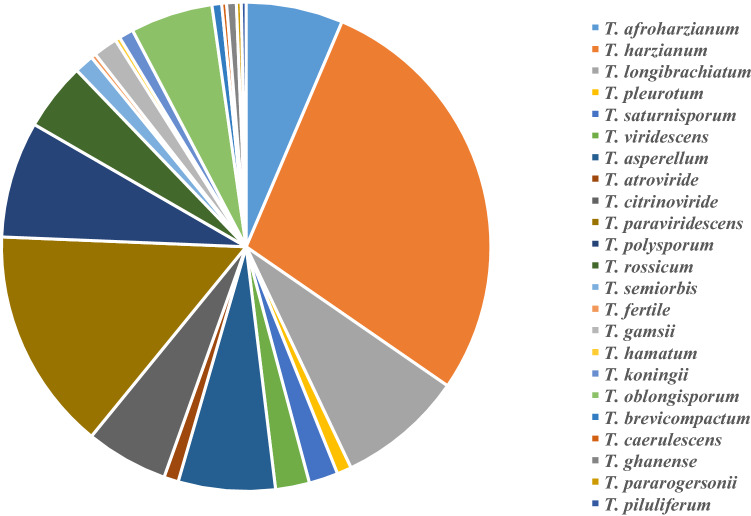
Biodiversity of *Trichoderma* spp. with 23 species from grassland and forest in Northern Xinjiang. 23 species: *T. harzianum* (88 strains), *T. paraviridescens* (46), *T. longibrachiatum* (26), *T. polysporum* (24), *T. asperellum* (20), *T. afroharzianum* (20), *T. oblongisporum* (17), *T. citrinoviride* (17), *T. rossicum* (14), *T. viridescens* (7), *T. saturnisporum* (6), *T. gamsii* (5), *T. semiorbis* (4), *T. pleurotum* (3), *T. koningii* (3), *T. atroviride* (3), *T. ghanense* (2), *T. brevicompactum* (2), *T. piluliferum* (1), *T. hamatum* (1), *T. pararogersonii* (1), *T. fertile* (1), and *T. caerulescens* (1)

The analysis was based on the 312 *Trichoderma* strains isolated from soil of selected regions in northern Xinjiang. A total of 212 soil samples (isolation rate = 33.28%) produced *Trichoderma* isolates from 634 varied soil samples, and 2,859 *Trichoderma* isolates (relative rate = 3%) were identified from 19,848 fungal colonies. The total biodiversity of *Trichoderma* spp. from grassland and forest in northern Xinjiang is shown in Fig. 2. A total of 23 species, including one new species record in China (*T. pararogersonii*), were recorded. The dominance value (Y) was 0.038 (> 0.02), indicating that the genus *Trichoderma* was dominant in soil samples. *T. harzianum* was the most abundant species (28.21%) followed by *T. paraviridescens* (14.74%).

The obtained data were used to calculate the Simpson’s biodiversity index (Dr), the Shannon-Weiner biodiversity index (H), the Pielou species evenness index (J), and Margalef’s abundance index (E) for each region (Supplemental Table 3). The highest species diversity and evenness were in the following order: Yili > Changji > Altay > Urumqi > Bayingolin. For the J index, Urumqi and Altay were characterized by lower homogeneity of species, while Yili, Bayingolin, and Changji were more homogenous. These results revealed that the grassland and forest ecosystem of northern Xinjiang had high diversity of *Trichoderma* spp. with optimum variation.

### Distribution of *Trichoderma* spp. in different regions

The distribution of *Trichoderma* strains in different regions is presented in supplemental Fig. 1. We found that the numbers of *Trichoderma* strains had a decreasing trend from north to south. The proportion and composition of *Trichoderma* species varied between different regions: Altay Prefecture had the largest number of *Trichoderma* species (17), with a slightly lower number (16) in Yili; Changji (ten) was in the middle, while Bayingolin and Urumqi had fewer species (seven and six, respectively). However, the proportion of strains obtained from Altay was the highest (182 strains, 58%), followed by Yili (48 strains, 15%), Changji (36 strains, 12%), Urumqi (31 strains, 10%), and Bayingolin (15 strains, 5%).

A total of 17 species were identified from Altay: *T. harzianum, T. paraviridescens, T. asperellum, T. citrinoviride, T. polysporum, T. oblongisporum, T. rossicum, T. saturnisporum, T. gamsii, T. longibrachiatum, T. viridescens, T. brevicompactum, T. afroharzianum, T. caerulescens, T. ghanense, T. pararogersonii,* and *T. piluliferum*. *Trichoderma* from Yili comprised 16 species: *T. harzianum, T. longibrachiatum, T. pleurotum, T. viridescens, T. asperellum, T. atroviride, T. citrinoviride, T. paraviridescens, T. polysporum, T. rossicum, T. semiorbis, T. fertile, T. gamsii, T. hamatum, T. koningii,* and *T. oblongisporum*. Ten species were found in Changji: *T. afroharzianum, T. harzianum, T. longibrachiatum, T. asperellum, T. atroviride, T. citrinoviride, T. paraviridescens, T. polysporum, T. rossicum,* and *T. semiorbis.* Seven species were found in Bayingolin: *T. harzianum, T. saturnisporum, T. asperellum, T. paraviridescens, T. polysporum,* and *T. koningii.* Six species were found in Urumqi: *T. afroharzianum, T. harzianum, T. longibrachiatum, T. pleurotum, T. saturnisporum,* and *T. viridescens.*(Supplemental Table 1).

Some of the species were unique to one region—*T. caerulescens*, *T. pararogersonii*, *T. brevicompactum*, and *T. piluliferum* were only collected from Altay; *T. fertile* and *T. hamatum* only from Yili; and *T. ghanense* only from Bayingolin. *T. harzianum*, however, was found in all regions and was the most widely distributed species. Another widely distributed species was *T. longibrachiatum*, which was distributed in all regions except Bayingolin. Among the 23 species described in this study, the *T. harzianum* complex (*T. harzianum* and *T. afroharzianum*) was the most abundant species (35% of all strains) with dominancy in Altay and Bayingolin. Other regions had a different dominant species: Urumqi and Yili’s dominant species were *T. longibrachiatum* and *T. polysporum*, respectively. Another very common species complex (17% of all strains) occurring in the five regions was *T. viridescens* (*T. viridescens* and *T. paraviridescens*). *T. longibrachiatum*, *T. polysporum*, and *T. asperellum* were distributed in four regions (Altay,Yili, Changji and Urumqi) with relatively lower proportions (8%, 8%, and 6%, respectively). The other species were represented by several strains and accounted for the minority of isolates (26% of all strains).

### Distribution of *Trichoderma* spp. in different ecosystems

The distribution of *Trichoderma* varied with ecosystems. According to the different ecological environments, the sampling sites can be divided into grassland and forest ecosystems. The grassland also included two sub-ecosystems—desert steppe and temperate steppe—while the forest ecosystem consisted of coniferous forest as well as coniferous and broadleaf mixed forest sub-ecosystems. There were 446 samples from the grassland ecosystem with 163 strains (52.24% of total strains) and 20 species of *Trichoderma*. A total of 188 samples were collected from the forest ecosystem with 149 strains (47.76% of total strains) and 16 species. The number of samples collected in the forest ecosystem was lower and the number of *Trichoderma* strains was lower than in the grassland ecosystem. However, the isolation frequency of *Trichoderma* in the forest ecosystem was 79.26%, which was significantly higher than that of grassland at 36.55%. Therefore, the forest ecosystem is the dominant ecosystem of *Trichoderma*, and it is more suitable for the survival and colonization of *Trichoderma* spp.

**Fig. 3:**
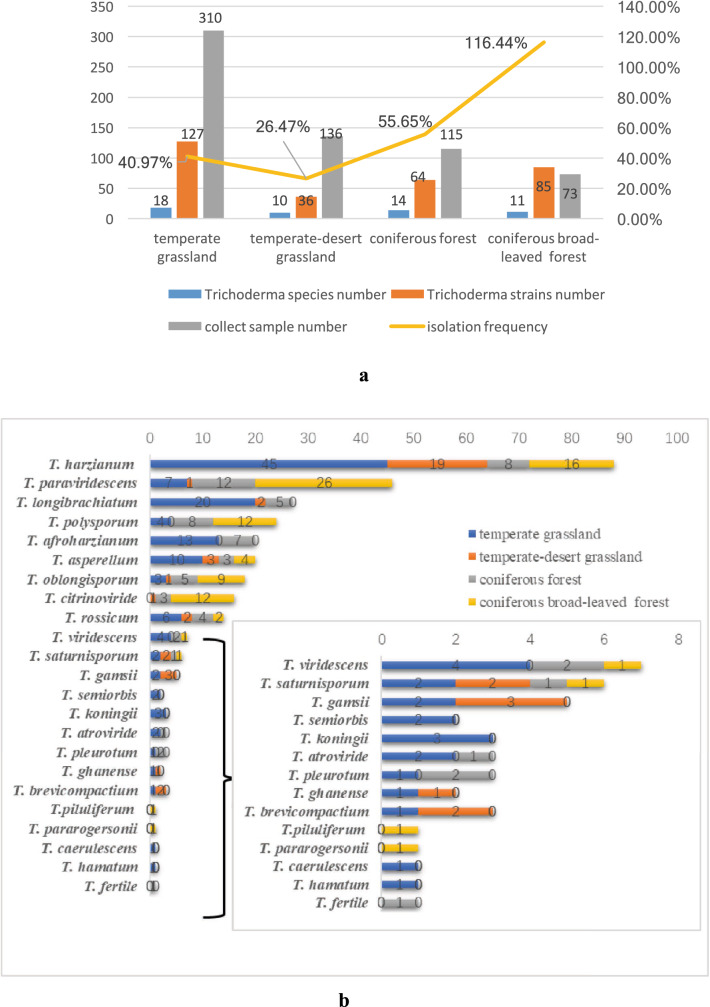
Distribution of *Trichoderma* species in different ecosystems. **a** The column curve picture for the positive detection of *Trichoderma* strains and species in different ecosystems. The numbers written on the bars correspond to the number of isolates in every ecosystem. The numbers written on the yellow line correspond to the frequency of *Trichoderma* in every ecosystem. **b** Distribution of *Trichoderma* species in different ecosystems. The inlaid box indicates the distributions of *T. viridescens, T. saturnisporum, T. gamsii, T. semiorbis, T. koningii, T. atroviride, T. pleurotum, T. ghanense, T. brevicompactum, T. piluliferum, T. pararogersonii, T. caerulescens, T. hamatum,* and *T. fertile* according to the different ecosystem. The numbers written on the bars correspond to the number of isolates in every ecosystem

In total, 312 *Trichoderma* strains were collected from the four ecosystems; the differences among them are shown in Fig. 3a. The highest species’ numbers were obtained from temperate grassland soil (18 species) isolated from 127 strains, followed by coniferous forest (14 species isolated from 64 strains), coniferous broadleaf forest (11 species isolated from 85 strains), and the temperate-desert grassland had the fewest species (ten species isolated from 36 strains). In addition, the order of isolation frequency of *Trichoderma* strains from soil samples from high to low is: coniferous broadleaf forest (116.44%), coniferous forest (55.65%), temperate grassland (40.97%), and temperate-desert grassland (26.47%).

The distribution of *Trichoderma* species is also related to the ecosystem (Fig. 3b). Some species like *T. harzianum*, *T. paraviridescens*, *T. asperellum*, *T. rossicum*, *T. oblongisporum* and *T. saturnisporum* were distributed in all four ecosystems; and some species were distributed in only one ecosystem, for example, *T. semiorbis*, *T. koningii*, *T. caerulescens*, and *T. hamatum* were only found in temperate grassland; *T. piluferum* and *T. paraogersonii* were only found in coniferous broadleaf forest; and *T. fertile* was only found in coniferous forest.

### Distribution of *Trichoderma* spp. at different altitudes

**Fig. 4:**
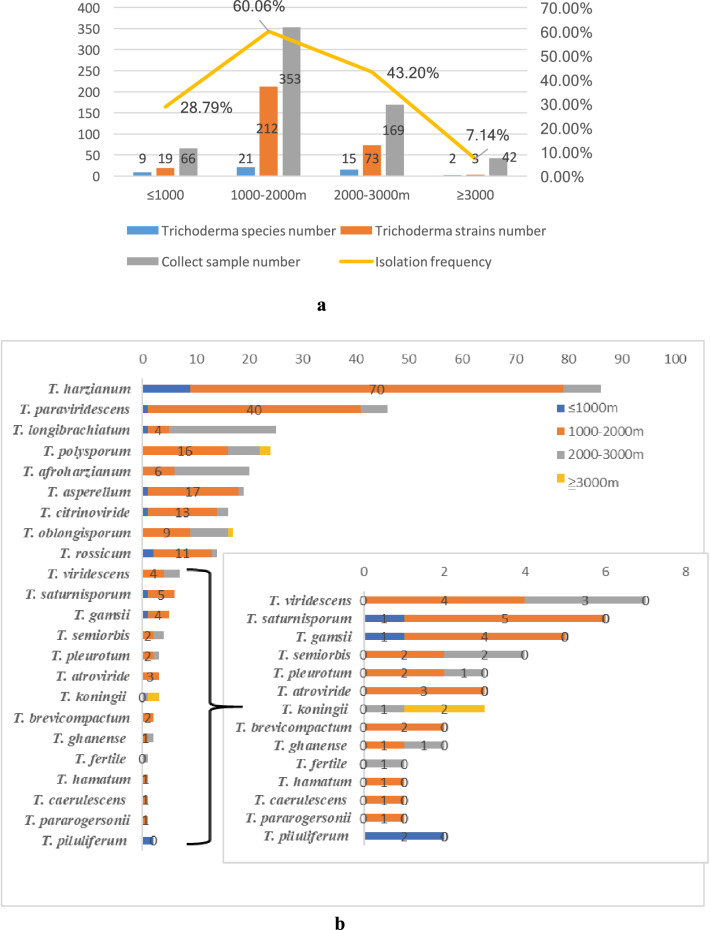
Distribution of *Trichoderma* species on different altitudes. **a** The column curve picture for the positive detection of *Trichoderma* strains and species on different altitudes. The numbers written on the bars correspond to the number of isolates in every ecosystem. The numbers written on the yellow line correspond to the isolate frequency of *Trichoderma* in every ecosystem. **b** Distribution of *Trichoderma* species on different altitudes. The inlaid box indicates the distributions of *T. viridescens, T. saturnisporum, T. gamsii, T. semiorbis, T. koningii, T. atroviride, T. pleurotum, T. ghanense, T. brevicompactum, T. piluliferum, T. pararogersonii, T. caerulescens, T. hamatum,* and *T. fertile* according to the different altitudes. The numbers written on the bars correspond to the number of isolates in every altitude

According to the altitude, all collection sites can be divided as follows: below 1000 m, 1000–2000 m, 2000 to 3000 m, and above 3000 m. There were significant differences in communities of *Trichoderma* species from soils collected at different altitudes (Fig. 4a). Twenty-one species of *Trichoderma* were isolated from 1000 to 2000 m, which covered most of the species described in this study, and the number of strains was the highest (212) at this altitude, accounting for 67.95% of all strains. At altitudes of 2000–3000 m, there were 15 species and 73 strains, the distribution of *Trichoderma* was dense, but the richness of *Trichoderma* spp. was reduced. At altitudes below 1000 m, only 66 soil samples were collected, but nine species and 19 strains of *Trichoderma* were isolated. However, at altitudes above 3000 m, there were 42 samples collected, fewer than below 1000 m, and only two species and three strains were isolated. According to the isolation frequency, the four altitude groups can be ranked from high to low as follows: 1000–2000 m, 2000–3000 m, below 1000 m, and above 3000 m. Based on these results, 1000–2000 m is a suitable living environment for *Trichoderma*, but above 3000 m might not be suitable for *Trichoderma* to survive in our selected sites.

In addition, the composition and distribution of *Trichoderma* at different altitudes were quite different (Fig. 4b). *T. harzianum* was distributed at all altitudes. The highest number of *T. harzianum* strains occurred between 1,000 and 2000 m, accounting for 80.46% of the total number of strains. The distribution of *T. paraviridescens* was similar to *T. harzianum*, with the largest distribution at altitudes of 1000–2000 m with 40 strains isolated, accounting for 86.96% of the total number of strains. *T. asperellum*, *T. citrinoviride*, and *T. rossicum* were distributed in the same way. *T. polysporum*, *T. afroharzianum, T. oblongisporum, T. viridescens, T. semiorbis, T. koningii, T. pleurotum*, and *T. ghanense* were not isolated at altitudes below 1000 m, indicating that higher altitudes may be more suitable for these species. The number of strains of *T. longibrachiatum* was the highest at altitudes between 2000 and 3000 m lower at 1000–2000 m, and lowest at altitudes below 1000 m. *T. caerulescens, T. piluliferum, T. pararogersonii*, and *T. hamatum* were isolated only at altitudes of 1000–2000 m, but the strains were fewer. At altitudes above 3000 m, only three strains were isolated: two of *T. oblongisporum* and one of *T. polysporum*.

### Principal components analysis of the distribution of *Trichoderma* spp

**Fig. 5:**
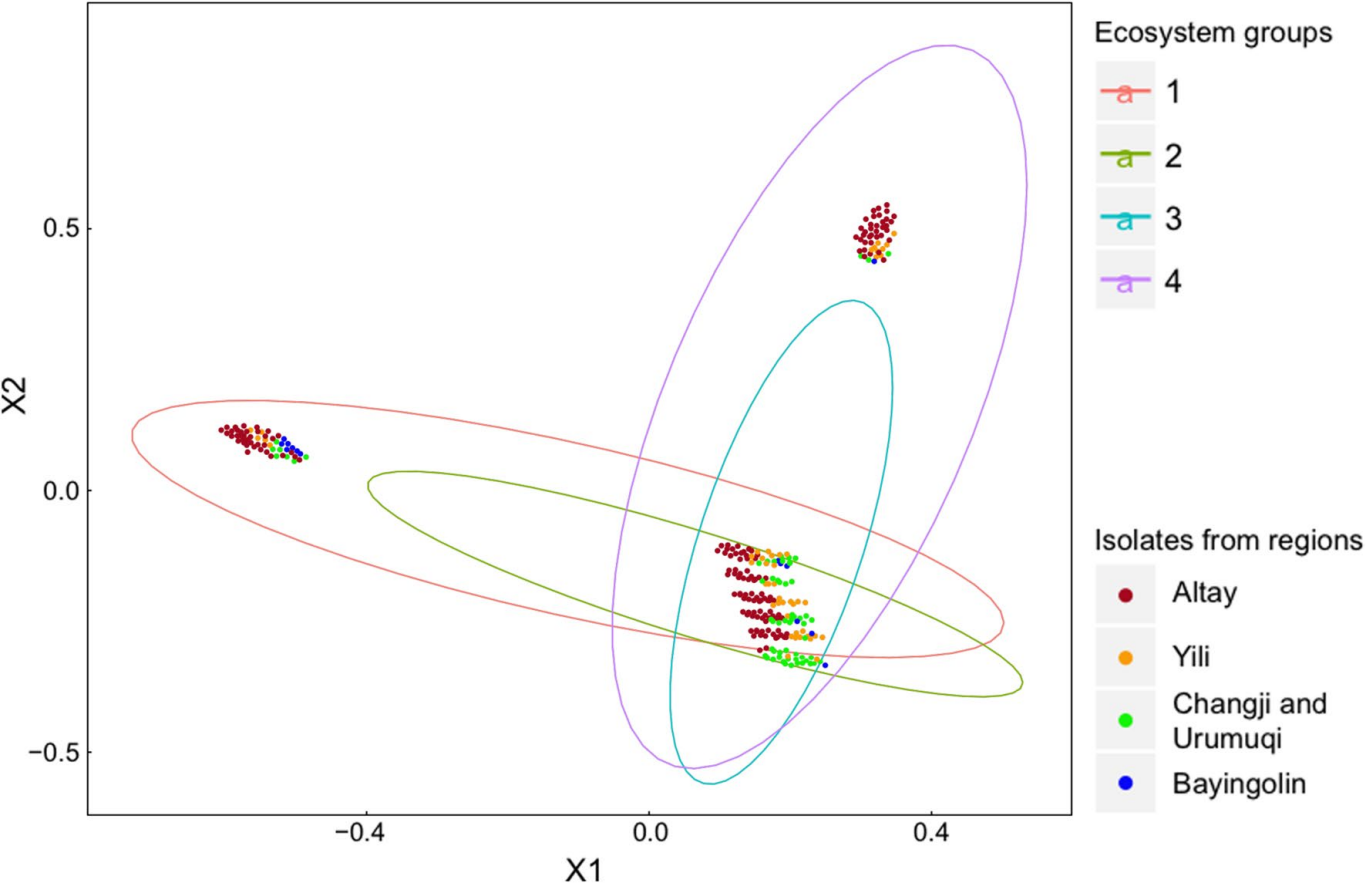
Principal Components Analysis (PCA) for all of the isolates of *Trichoderma* spp. from Northern Xinjiang by the R package vegan. Ecosystem groups: 1. desert steppe; 2. temperate steppe; 3. coniferous forest; 4. coniferous and broadleaf mixed forest. The colors of isolates from location regions: Altay (deep red); Yili (orange); 3. Changji and Urumuqi (bright green); 4. Bayingolin (blue)

All of the data for isolates of *Trichoderma* spp. were analyzed using principal components analysis (PCA) in R package vegan. The results showed us the ecosystem is the most dominant impact factor for the distribution of *Trichoderma* spp. among longitude, latitude, altitude and ecosystems (Fig. 5). The grassland and forest ecosystems were contained two different ecosystems, respectively, the grassland had two sub-ecosystems as desert steppe and temperate steppe, while forest had two sub-ecosystems with coniferous forest as well as coniferous and broadleaf mixed forest. In the same ecosystem or had two focus distribution areas, which were induced by altitude.

## Discussion

In this study, we examined the biodiversity of *Trichoderma* associated with collection region, ecosystem, and altitude from grassland and forest in northern Xinjiang. Several studies have investigated the biodiversity of *Trichoderma* in different provinces of China(Jiang et al. 2016; Saravanakumar et al. 2016; Sun et al. 2012; Zhang et al. 2005a, b); however, little attention was paid to Xinjiang. In 2014, 2015, and 2016, 634 soil samples were collected from grassland and forest ecosystems in five major regions of northern Xinjiang, a total of 2,859 isolates were obtained, 312 strains were classified, and 23 species were identified, including a new species record in China (*T. pararogersonii*). Compared to studies on the biodiversity in other countries and regions, like Poland(Błaszczyk et al. 2011), Central Europe(Błaszczyk et al. 2016), and the Indo-Burma Biodiversity Hot Spot(Kamala et al. 2015), there were almost twice the number of *Trichoderma* species (23) detected in our study. In addition, our results showed that *Trichoderma* distributions were remarkably diversified: the dominant species and proportion of each species were significantly different in different regions, and such a varied distribution might be associated with differences in ecological environments such as ecosystem type and altitude of the large land mass in Xinjiang.

The composition and proportion of *Trichoderma* species and strains were distinct among different regions. Geographic distance is the dominant factor driving variation in fungal diversity at a regional scale (1000–4000 km)(Wu et al. 2013), which is consistent with our research. In this study, the total number of *Trichoderma* strains in northern Xinjiang varied from north to south. Altay had 17 species and had the largest number of *Trichoderma* strains (182), followed by Yili (16 species, 48 strains), Changji (10 species, 36 strains), Bayingolin (7 species, 15 strains) and Urumqi (6 species, 31 strains). Fungal diversity is related to vegetation zones and environmental factors (Zachow et al. 2009a, b), which might account for this distinct distribution. The ecological environment of Altay is diverse, including four ecological types: temperate grassland, temperate-desert grassland, coniferous forest, and coniferous broadleaf forest. The vegetation cover is also dense, which may have led to the high isolation frequency of *Trichoderma*. The ecological environment of some collection sites in Bayingolin is very poor, with ground cover of only a small amount of desert vegetation and soil types that are mostly sandy soil, but other parts of the region have more favorable ecological environments and dense vegetation cover. However, the high altitude of Bayingolin, which can reach more than 3000 m, may also negatively affect the colonization of *Trichoderma*.

The distribution of *Trichoderma* varies with changes in the ecosystem. Researchers have reported that fungal communities are associated with distinct plant species(de Souza Sebastianes et al. 2013), vegetation cover density, edaphic factors(Birhane et al. 2018), soil age, and ecosystem development(Courty et al. 2018). In this study, the vegetation types of forest ecosystems in northern Xinjiang were diverse, the soil was more fertile, and *Trichoderma* was widely distributed. Although the overall sampling number (188) in the forest was less than that of the grassland (446), the isolation frequency of *Trichoderma* was higher (79.26% and 36.55%, respectively). Therefore, the forest ecosystem is dominant for *Trichoderma* and is more suitable for survival and colonization of *Trichoderma*. This indicated that *Trichoderma* has an environmental preference, consistent with previous studies(Błaszczyk et al. 2011; Friedl and Druzhinina, 2011; Yu et al. 2010). Similarly, forest ecosystems subjected to natural dynamics for a longer period support a more diverse fungal community(Pioli et al. 2018).

Limited diversity of *Trichoderma* was found in temperate-desert grassland, which may be attributed to the poor ecological environment. In these ecosystems, there is only desert vegetation, such as shuttle, caraway grass and red willow (*Tamirix chinensis*), and soil types are sandy and sandy loam, which may be detrimental to the colonization of *Trichoderma*. In the forest ecosystem, the number of *Trichoderma* strains isolated from coniferous broadleaf forest (85) was higher than that of coniferous forest (64), while the number of *Trichoderma* species (14) obtained from coniferous forest was higher than that of coniferous broadleaf forest (11). This may be because more soil samples were collected in the coniferous forest, with a wider range of collection, including parts of Urumqi, Changji, Yili and Altay, but the only coniferous broadleaf forest collection site was in Kanas Nature Reserve in Altay. In addition, the isolation frequency of coniferous broadleaf forest was higher, which might be attributed to the better ecological environment (mean temperature, maximum and minimum relative humidity, cumulative rainfall) and higher vegetation coverage, or may be associated with the soil moisture, pH, and oxygen content.

The numbers of species and strains isolated from different altitudes were highly variable. Yu et al. investigated the diversity of *Trichoderma* in Guangxi and found that altitude had a minimal effect on richness(Yu et al. 2012). Their investigation in a forest in Guangxi was conducted at altitudes between 100-2,100 m, which was lower compared to our altitude range of 325-3,547 m, and Guangxi is a rainy region. Other recent evidence has revealed that community composition and diversity patterns of some soil microbiota correlate strongly with altitude: we found that the distribution of *Trichoderma* was related to the altitude of the sampling site based on a decline of quantity and species in different kinds of fungi (Shi et al. 2014; Dong et al. 2004) with altitudes above 3000 m or below 1000 m. The order of the *Trichoderma* diversity at different altitudes is: 2000–3000 m, 1000–2000 m, below 1000 m, and above 3000 m. This trend was in accordance with reports from Yungas forests, in which the total fungal diversity did not decrease significantly with increasing elevation from 400 to 2160 m a.s.l (Geml et al. 2014), but contrary to Yu et al. (2012). who found that fungal species richness declined gradually with increasing elevation. Our results indicated that altitudes under 3000 m was a suitable living environment, but above 3000 m was not suitable for *Trichoderma* to survive, which might be attributed to low temperature. Gomes et al. demonstrated that fungal community structure was also driven by mean temperatures at 10 (for endophytes) or 20 (for epiphytes) days before the sampling date (Gomes et al. 2018). The dominant species was different in every altitude gradient, indicating that lower altitudes should be more suitable for *T. harzianum*, while higher altitudes are more suitable for *T. longibrachiatum* and *T. polysporum*.

Here and in a previous study, *T. harzianum* was the dominant taxon(Jaklitsch and Voglmayr 2015; Kubicek et al. 2008). *T. harzianum* is the most commonly reported species in the genus, occurring in diverse ecosystems and ecological niches. High genetic diversity may contribute to the higher abundance of *T. harzianum*: we found six haplotypes in this study and it has been reported that *T. harzianum* is a species complex (Chaverri et al. 2015). We only identified this species according to the ITS region, so there might be new species within the species complex. The second most dominant taxon in Xinjiang was *T. paraviridescens*, which belongs to the *T. viridescens* complex that contains 13 species (Jaklitsch et al. 2013). In contrast, Wu et al. found that *T. viridescens* had the smallest communities in the soils of northwestern China (Wu et al. 2017), indicating that *T. viridescens* may only be distributed in Xinjiang, especially in Altay.

*T. pararogersonii* was first reported from China in this paper. Previously, this species was only reported by Jaklitsch et al. to be distributed in Mediterranean Europe (Jaklitsch et al. 2013), here we expanded its distribution range. There is no report of its ITS sequence, so we identified it by analyzing the sequences of *tef* and *rpb*2, which had similarities of 100% with the reported sequence of *T. pararogersonii* from GenBank.

To our knowledge, this is the first report that analyzes *Trichoderma* biodiversity from grassland and forest ecosystems in Xinjiang Uygur Autonomous Region, China, and this study includes the identification of a large number of *Trichoderma* strains based on molecular biology. The data generated in this study reveals a great reservoir of *Trichoderma* genetic diversity in the Chinese forests and grasslands and highlights substantial differences between *Trichoderma* communities from distinct sampling regions, ecosystems, and altitudes. This work could be important in the future to detect possible valuable *Trichoderma* resources in local environments. In the meantime, new experiments should be performed to better understand the biocontrol ability of *Trichoderma* to select some strains that can be used as inoculants for plant growth and health promotion, such as fungicide applications and/or introduction of biological control agents.

## Electronic supplementary material

Below is the link to the electronic supplementary material.

Supplementary material 1 (DOCX 758 kb)

Supplementary material 2 (PDF 62 kb)

## Abbreviations

PDA: Potato dextrose agar
SNA: Synthetic low nutrient agar
CMD: Corn meal dextrose agar
NCBI: National Center for Biotechnology Information
BLAST: Basic local alignment search tool
PCR: Polymerase Chain Reaction
Bp: Base pair
ddH_2_O: Double Distilled H_2_O
TEF1-α: Transcription Elongation Factor 1 α
RPB2: RNA Polymerase II second largest subunit
ITS: Internal Transcribed Spacer
F: *Fusarium oxysporum*
R: *Rhizoctonia solani*
B: *Botrytis cinerea*
MS: Murashige and Skoog
