# Supplementary material for: Biodiversity of Trichoderma from grassland and forest ecosystems in Northern Xinjiang, China
Source: 3 Biotech. 2020 Jul 30;10(8):362. doi: 10.1007/s13205-020-02301-6 (PMC7392985; doi:10.1007/s13205-020-02301-6)

**Supplemental Tab. 1** Supplemental Tab 1. Distribution of *Trichoderma* in northern Xinjiang. This table indicates the distribution of *Trichoderma* in the five main regions of northern Xinjiang (Urumqi, Changji, Yili, Altay, and Bayingolin).

| Trichoderma species | Urumqi | Changji | Yili | Altay | Bayingolin | Total strain number |
| --- | --- | --- | --- | --- | --- | --- |
| *T. harzianum* | 3 | 4 | 8 | 66 | 7 | 88 |
| *T. paraviridescens* | 0 | 3 | 4 | 37 | 2 | 46 |
| *T.longibrachiatum* | 16 | 6 | 2 | 2 | 0 | 26 |
| *T. polysporum* | 0 | 1 | 9 | 13 | 1 | 24 |
| *T. afroharzianum* | 8 | 11 | 0 | 1 | 0 | 20 |
| *T. asperellum* | 0 | 1 | 1 | 17 | 1 | 20 |
| *T. citrinoviride* | 0 | 1 | 3 | 13 | 0 | 17 |
| *T. oblongisporum* | 0 | 0 | 8 | 9 | 0 | 17 |
| *T. rossicum* | 0 | 5 | 1 | 8 | 0 | 14 |
| *T. viridescens* | 1 | 0 | 4 | 2 | 0 | 7 |
| *T. saturnisporum* | 1 | 0 | 0 | 4 | 1 | 6 |
| *T. gamsii* | 0 | 0 | 1 | 4 | 0 | 5 |
| *T. semiorbis* | 0 | 3 | 1 | 0 | 0 | 4 |
| *T. pleurotum* | 2 | 0 | 1 | 0 | 0 | 3 |
| *T. atroviride* | 0 | 1 | 2 | 0 | 0 | 3 |
| *T. koningii* | 0 | 0 | 2 | 0 | 1 | 3 |
| *T.brevicompactum* | 0 | 0 | 0 | 2 | 0 | 2 |
| *T. ghanense* | 0 | 0 | 0 | 1 | 1 | 2 |
| *T. fertile* | 0 | 0 | 1 | 0 | 0 | 1 |
| *T. hamatum* | 0 | 0 | 1 | 0 | 0 | 1 |
| *T. caerulescens* | 0 | 0 | 0 | 1 | 0 | 1 |
| *T. pararogersonii* | 0 | 0 | 0 | 1 | 0 | 1 |
| *T. piluliferum* | 0 | 0 | 0 | 1 | 0 | 1 |

**Supplemental Table 2. Haplotypes (51) of *Trichoderma* identified in this study**

| **Haplotype** | **Species name** | **Strain number** | **Representative strains** | |
| --- | --- | --- | --- | --- |
|  |  |  | **Code** | **GenBank Accession** |
| AF1 | *T. afroharzianum* | 20 | CTCCSJ-G-HB40296 | KY764838 |
| AP1 | *T. asperellum* | 1 | CTCCSJ-F-ZY40342 | KY764822 |
| AP2 | *T. asperellum* | 16 | CTCCSJ-G-QT40303 | KY764851 |
| AP3 | *T. asperellum* | 1 | CTCCSJ-F-ZY40590 | KY750362 |
| AP4 | *T. asperellum* | 1 | CTCCSJ-F-KZ40636 | KY750421 |
| AP5 | *T. asperellum* | 1 | CTCCSJ-G-QT40835 | KY750493 |
| AV1 | *T. atroviride* | 1 | CTCCSJ-G-HB40436 | KY764874 |
| AV2 | *T. atroviride* | 2 | CTCCSJ-F-ZY40745 | KY750328 |
| CL1 | *T. caerulescens* | 1 | CTCCSJ-G-HB40837 | KY750479 |
| CV1 | *T. citrinoviride* | 8 | CTCCSJ-F-ZY40023 | KY764888 |
| CV2 | *T. citrinoviride* | 7 | CTCCSJ-F-KY40021 | KY764890 |
| CV3 | *T. citrinoviride* | 1 | CTCCSJ-F-KZ40703 | KY750454 |
| FT1 | *T. fertile* | 1 | CTCCSJ-F-KZ40847 | KY750508 |
| GM1 | *T. gamsii* | 5 | CTCCSJ-G-HB40456 | KY764863 |
| GH1 | *T. ghanense* | 2 | CTCCSJ-G-HB40024 | KY764887 |
| HM1 | *T. hamatum* | 1 | CTCCSJ-G-QT40427 | KY764884 |
| HZ1 | *T. harzianum* | 40 | CTCCSJ-G-QT40001 | KY764853 |
| HZ2 | *T. harzianum* | 4 | CTCCSJ-G-HB40437 | KY764875 |
| HZ3 | *T. harzianum* | 1 | CTCCSJ-G-QT40003 | KY764911 |
| HZ4 | *T. harzianum* | 1 | CTCCSJ-G-QT40443 | KY764914 |
| HZ5 | *T. harzianum* | 42 | CTCCSJ-G-QT40002 | KY764854 |
| KN1 | *T. koningii* | 3 | CTCCSJ-G-HB40432 | KY764897 |
| LB1 | *T. longibrachiatum* | 24 | CTCCSJ-G-HB40189 | KY764837 |
| LB2 | *T. longibrachiatum* | 1 | CTCCSJ-G-HB40276 | KY764813 |
| LB3 | *T. longibrachiatum* | 1 | CTCCSJ-G-QT40192 | KY764850 |
| LB4 | *T. longibrachiatum* | 1 | CTCCSJ-G-QT40274 | KY764852 |
| OS1 | *T. oblongisporum* | 14 | CTCCSJ-F-KZ40818 | KY750457 |
| OS2 | *T. oblongisporum* | 1 | CTCCSJ-F-KZ40826 | KY750468 |
| OS3 | *T. oblongisporum* | 2 | CTCCSJ-G-HB40839 | KY750512 |
| PRG1 | *T. pararogersonii* | 1 | CTCCSJ-F-KZ40688 | KY750455 |
| PV1 | *T. paraviridescens* | 46 | CTCCSJ-F-ZY40742 | KY750331 |
| PF1 | *T. piluliferum* | 1 | CTCCSJ-F-KZ40801 | KY750473 |
| PL1 | *T. pleurotum* | 3 | CTCCSJ-F-ZY40450 | KY764870 |
| PS1 | *T. polysporum* | 15 | CTCCSJ-F-ZY40741 | KY750323 |
| PS2 | *T. polysporum* | 2 | CTCCSJ-F-KZ40799 | KY750488 |
| PS3 | *T. polysporum* | 1 | CTCCSJ-G-HB40844 | KY750507 |
| PS4 | *T. polysporum* | 1 | CTCCSJ-F-KZ40848 | KY750509 |
| PS5 | *T. polysporum* | 1 | CTCCSJ-G-JK40726 | KY750510 |
| PS6 | *T. polysporum* | 4 | CTCCSJ-F-KZ40694 | KY750478 |
| RS1 | *T. rossicum* | 4 | CTCCSJ-F-ZY40498 | KY764840 |
| RS2 | *T. rossicum* | 6 | CTCCSJ-G-HB40426 | KY764880 |
| RS3 | *T. rossicum* | 1 | CTCCSJ-G-QT40602 | KY750358 |
| RS4 | *T. rossicum* | 1 | PDA X100-1（2016） | KY750334 |
| RS5 | *T. rossicum* | 1 | CTCCSJ-G-QT40748 | KY750352 |
| RS6 | *T. rossicum* | 1 | CTCCSJ-F-KZ40775 | KY750355 |
| ST1 | *T. saturnisporum* | 4 | CTCCSJ-F-ZY40006 | KY764857 |
| ST2 | *T. saturnisporum* | 1 | CTCCSJ-G-JK40418 | KY764867 |
| ST3 | *T. saturnisporum* | 1 | PDA X072-5（2016） | KY750402 |
| SM1 | *T. semiorbis* | 4 | CTCCSJ-F-ZY40406 | KY764821 |
| BV1 | *T. brevicompactum* | 2 | CTCCSJ-G-JK40771 | KY750391 |
| VD1 | *T. viridescens* | 7 | CTCCSJ-G-HB40322 | KY764814 |

**Supplemental Table 3. Statistical analysis of *Trichoderma* diversity in five main regions of Northern Xinjiang**

| **Ecological indices** | **Urumqi** | **Changji** | **Yili** | **Altay** | **Bayingolin** |
| --- | --- | --- | --- | --- | --- |
| Simpson index (Dr) | 3.06 | 6.85 | 10.25 | 5.18 | 4.77 |
| Shannon-Weiner index（H） | 1.32 | 1.99 | 2.46 | 2.04 | 1.71 |
| Margalef index (E) | 1.46 | 2.51 | 3.87 | 3.07 | 2.22 |
| Pielou evenness index（J） | 0.73 | 0.86 | 0.89 | 0.72 | 0.88 |

**Supplemental Fig. 1**


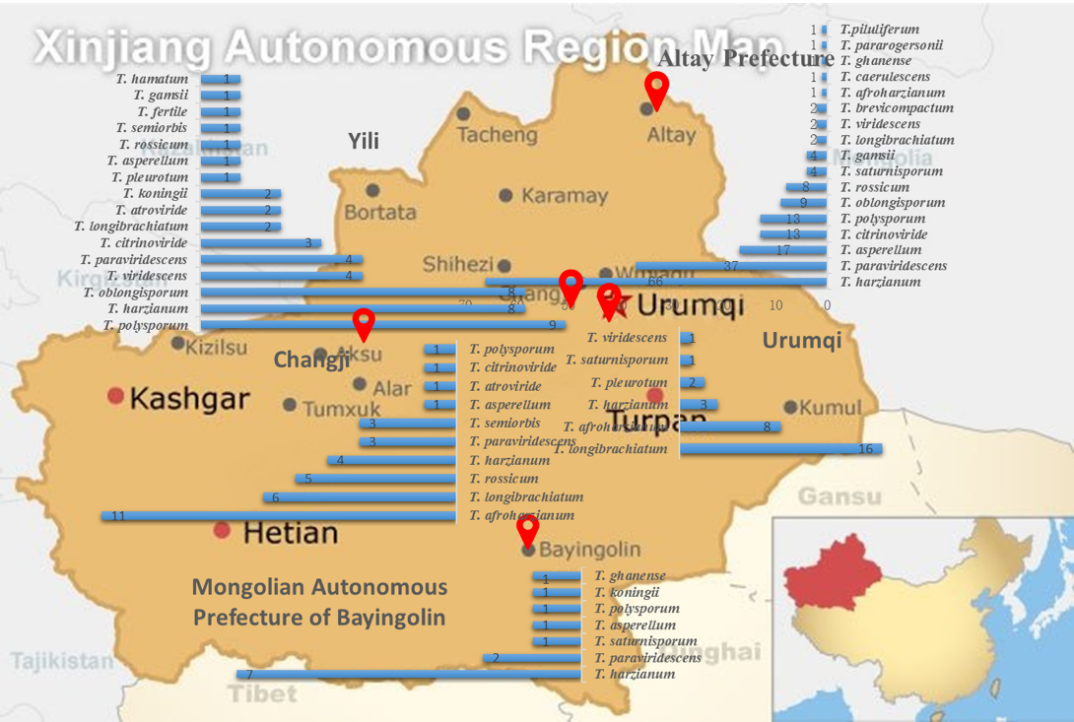

Supplement: Supplementary file 1 — Supplementary material 1 (DOCX 758 kb) [file 13205_2020_2301_MOESM1_ESM.docx]
