# Supplementary material for: Biodiversity of Trichoderma from grassland and forest ecosystems in Northern Xinjiang, China
Source: 3 Biotech. 2020 Jul 30;10(8):362. doi: 10.1007/s13205-020-02301-6 (PMC7392985; doi:10.1007/s13205-020-02301-6)
Supplement: Supplementary file 2 — Supplementary material 2 (PDF 62 kb) [file 13205_2020_2301_MOESM2_ESM.pdf]

Sample	Altitude	Ecosystem	Longitude	Latitude	Region	Species
ALBRC1	775	1	87.0508611	48.1686667	1	T.brevicompactum
ALBRC2	775	1	87.0508611	48.1686667	1	T.brevicompactum
ALBRC3	775	1	87.0508611	48.1686667	1	T.gamsii
ALBRC4	775	1	87.0508611	48.1686667	1	T.harzianum
ALBRC5	775	1	87.0508611	48.1686667	1	T.harzianum
ALBRC6	1204	1	87.0211389	48.1896111	1	T.harzianum
ALBRC7	1204	1	87.0211389	48.1896111	1	T.harzianum
ALBRC9	775	1	87.0508611	48.1686667	1	T.paraviridescens
ALBRC10	775	1	87.0508611	48.1686667	1	T.rossicum
ALBRC11	775	1	87.0508611	48.1686667	1	T.rossicum
ALBRC12	775	1	87.0508611	48.1686667	1	T.saturnisporum
AFKK1	1207	2	89.8516667	47.2108889	1	T.gamsii
AFKK2	1256	2	89.8707778	47.208	1	T.gamsii
AFKK3	1258	2	89.8692222	47.2089444	1	T.harzianum
AFKK4	1302	2	89.8741944	47.20075	1	T.harzianum
AFKK5	1244	2	89.8688889	47.2091944	1	T.asperellum
AFKK6	1258	2	89.8692222	47.2089444	1	T.asperellum
AFKK7	1258	2	89.8692222	47.2089444	1	T.asperellum
AFKK8	1299	2	89.8738611	47.2025556	1	T.asperellum
AFKK9	1302	2	89.8741944	47.20075	1	T.asperellum
AFKK10	1207	2	89.8516667	47.2108889	1	T.harzianum
AFKK11	1218	2	89.8604444	47.2073611	1	T.harzianum
AFKK12	1218	2	89.8604444	47.2073611	1	T.harzianum
AFKK13	1210	2	89.8607778	47.2076667	1	T.harzianum
AFKK14	1210	2	89.8607778	47.2076667	1	T.harzianum
AFKK15	1244	2	89.8688889	47.2091944	1	T.harzianum
AFKK16	1256	2	89.8707778	47.208	1	T.harzianum
AFKK17	1256	2	89.8707778	47.208	1	T.harzianum
AFKK18	1299	2	89.8738611	47.2025556	1	T.harzianum
AFKK19	1299	2	89.8738611	47.2025556	1	T.harzianum
AFKK20	1299	2	89.8738611	47.2025556	1	T.harzianum
AFKK21	1302	2	89.8741944	47.20075	1	T.harzianum
AFKK25	1210	2	89.8607778	47.2076667	1	T.saturnisporum
AFKK26	1210	2	89.8607778	47.2076667	1	T.saturnisporum
AFKK27	1404	3	89.8764167	47.1956389	1	T.asperellum
AFKK28	1392	3	89.8763889	47.1958333	1	T.harzianum
AFKK29	1380	3	89.8763611	47.1957778	1	T.harzianum
AFKK30	1404	3	89.8764167	47.1956389	1	T.harzianum
AFKK31	1408	3	89.8764167	47.1956389	1	T.harzianum
AFKK32	1401	3	89.8761944	47.1955833	1	T.harzianum
AFKK33	1370	3	89.8761389	47.1955556	1	T.harzianum
AFKK34	1404	3	89.8764167	47.1956389	1	T.paraviridescens

AFKK35	1408	3	89.8764167	47.1956389	1	T.paraviridescens
AFKK36	1401	3	89.8761944	47.1955833	1	T.paraviridescens
AFKK37	1370	3	89.8761389	47.1955556	1	T.paraviridescens
AFKK39	1370	3	89.8761389	47.1955556	1	T.rossicum
AFKK40	1379	3	89.87675	47.1961667	1	T.paraviridescens
AFKK41	1404	3	89.8764167	47.1956389	1	T.paraviridescens
AFKK42	1443	3	89.8761944	47.1955833	1	T.paraviridescens
ALKNS1	1365	2	87.0515278	48.6198611	1	T.asperellum
ALKNS2	1361	2	87.0516944	48.6194722	1	T.asperellum
ALKNS3	1367	2	87.0519444	48.6194167	1	T.asperellum
ALKNS4	1386	2	87.02475	48.7086667	1	T.asperellum
ALKNS5	1363	2	87.0268611	48.7149722	1	T.asperellum
ALKNS6	1365	2	87.0515278	48.6198611	1	T.harzianum
ALKNS7	1365	2	87.0515278	48.6198611	1	T.harzianum
ALKNS8	1365	2	87.0515278	48.6198611	1	T.harzianum
ALKNS9	1361	2	87.0516944	48.6194722	1	T.harzianum
ALKNS10	1367	2	87.0519444	48.6194167	1	T.harzianum
ALKNS11	1386	2	87.02475	48.7086667	1	T.harzianum
ALKNS12	1384	2	87.0270833	48.71175	1	T.harzianum
ALKNS13	1384	2	87.0270833	48.71175	1	T.harzianum
ALKNS14	1391	2	87.0300833	48.7148333	1	T.harzianum
ALKNS15	1402	2	87.0317778	48.7145833	1	T.harzianum
ALKNS16	1408	2	87.0340833	48.7111389	1	T.harzianum
ALKNS17	1407	2	87.0342222	48.7100833	1	T.harzianum
ALKNS18	1367	2	87.0519444	48.6194167	1	T.harzianum
ALKNS19	1384	2	87.0270833	48.71175	1	T.paraviridescens
ALKNS20	1391	2	87.0300833	48.7148333	1	T.paraviridescens
ALKNS21	1367	2	87.0519444	48.6194167	1	T.paraviridescens
ALKNS22	1407	2	87.0342222	48.7100833	1	T.paraviridescens
ALKNS23	1363	2	87.0268611	48.7149722	1	T.paraviridescens
ALKNS24	1367	2	87.0519444	48.6194167	1	T.rossicum
ALKNS100	1398	4	87.0277778	48.7135833	1	T.polysporum
ALKNS101	1398	4	87.0277778	48.7135833	1	T.polysporum
ALKNS102	1398	4	87.0277778	48.7135833	1	T.polysporum
ALKNS103	1374	4	87.0269167	48.7028611	1	T.polysporum
ALKNS104	1370	4	87.027	48.70325	1	T.polysporum
ALKNS105	1350	4	87.0511944	48.6198056	1	T.rossicum
ALKNS106	1331	4	87.0503611	48.6185556	1	T.rossicum
ALKNS27	1400	4	87.0243611	48.7097222	1	T.citrinoviride
ALKNS28	1374	4	87.0223889	48.7121111	1	T.citrinoviride
ALKNS29	1376	4	87.0261111	48.7060556	1	T.citrinoviride
ALKNS30	1399	4	87.0243611	48.7097222	1	T.citrinoviride
ALKNS31	1373	4	87.0258056	48.7059167	1	T.citrinoviride
ALKNS32	1399	4	87.0243611	48.7097222	1	T.saturnisporum
ALKNS33	1383	4	87.0224444	48.7098333	1	T.viridescens

ALKNS34	1331	4	87.0503611	48.6185556	1	T.asperellum
ALKNS35	1331	4	87.0503611	48.6185556	1	T.asperellum
ALKNS36	1375	4	87.0250556	48.7139722	1	T.asperellum
ALKNS37	1331	4	87.0503611	48.6185556	1	T.citrinoviride
ALKNS38	1375	4	87.0250556	48.7139722	1	T.citrinoviride
ALKNS39	1375	4	87.0250556	48.7139722	1	T.citrinoviride
ALKNS40	1375	4	87.0250556	48.7139722	1	T.citrinoviride
ALKNS41	1404	4	87.0348889	48.7094167	1	T.citrinoviride
ALKNS42	1375	4	87.0250556	48.7139722	1	T.citrinoviride
ALKNS43	1350	4	87.0511944	48.6198056	1	T.harzianum
ALKNS44	1350	4	87.0511944	48.6198056	1	T.harzianum
ALKNS45	1350	4	87.0511944	48.6198056	1	T.harzianum
ALKNS46	1343	4	87.0506389	48.6188056	1	T.harzianum
ALKNS47	1331	4	87.0503611	48.6185556	1	T.harzianum
ALKNS48	1319	4	87.0497778	48.6184167	1	T.harzianum
ALKNS49	1393	4	87.0260833	48.7098611	1	T.harzianum
ALKNS50	1393	4	87.0260833	48.7098611	1	T.harzianum
ALKNS51	1393	4	87.0269444	48.7130556	1	T.harzianum
ALKNS52	1412	4	87.0340278	48.7114444	1	T.harzianum
ALKNS53	1384	4	87.0275556	48.71175	1	T.harzianum
ALKNS54	1393	4	87.0269444	48.7130556	1	T.harzianum
ALKNS55	1411	4	87.0336111	48.7129167	1	T.harzianum
ALKNS56	1412	4	87.0340278	48.7114444	1	T.harzianum
ALKNS57	1375	4	87.0250556	48.7139722	1	T.harzianum
ALKNS58	1404	4	87.0348889	48.7094167	1	T.harzianum
ALKNS59	1368	4	87.0268889	48.7036111	1	T.oblongisporum
ALKNS60	1368	4	87.0268889	48.7036111	1	T.oblongisporum
ALKNS61	1368	4	87.0268889	48.7036111	1	T.oblongisporum
ALKNS62	1368	4	87.0268889	48.7036111	1	T.oblongisporum
ALKNS63	1368	4	87.0273333	48.7016667	1	T.oblongisporum
ALKNS64	1368	4	87.0268889	48.7036111	1	T.oblongisporum
ALKNS65	1368	4	87.0268889	48.7036111	1	T.oblongisporum
ALKNS66	1382	4	87.0263889	48.7145278	1	T.oblongisporum
ALKNS67	1382	4	87.0263889	48.7145278	1	T.oblongisporum
ALKNS68	1386	4	87.0339444	48.6949722	1	T.pararogersonii
ALKNS69	1350	4	87.0511944	48.6198056	1	T.paraviridescens
ALKNS70	1331	4	87.0503611	48.6185556	1	T.paraviridescens
ALKNS71	1331	4	87.0503611	48.6185556	1	T.paraviridescens
ALKNS72	1319	4	87.0497778	48.6184167	1	T.paraviridescens
ALKNS73	1393	4	87.0260833	48.7098611	1	T.paraviridescens
ALKNS74	1393	4	87.0269444	48.7130556	1	T.paraviridescens
ALKNS75	1389	4	87.0283333	48.7135	1	T.paraviridescens
ALKNS76	1412	4	87.0340278	48.7114444	1	T.paraviridescens
ALKNS77	1405	4	87.0356111	48.7084444	1	T.paraviridescens
ALKNS78	1405	4	87.0356111	48.7084444	1	T.paraviridescens

ALKNS79	1405	4	87.0354722	48.7065833	1	T.paraviridescens
ALKNS80	1386	4	87.0339444	48.6949722	1	T.paraviridescens
ALKNS81	1363	4	87.0283889	48.7003611	1	T.paraviridescens
ALKNS82	1368	4	87.0273333	48.7016667	1	T.paraviridescens
ALKNS83	1370	4	87.027	48.70325	1	T.paraviridescens
ALKNS84	1370	4	87.027	48.70325	1	T.paraviridescens
ALKNS85	1343	4	87.0506389	48.6188056	1	T.paraviridescens
ALKNS86	1393	4	87.0260833	48.7098611	1	T.paraviridescens
ALKNS87	1393	4	87.0260833	48.7098611	1	T.paraviridescens
ALKNS88	1394	4	87.0258889	48.7100278	1	T.paraviridescens
ALKNS89	1384	4	87.0275556	48.71175	1	T.paraviridescens
ALKNS90	1393	4	87.0269444	48.7130556	1	T.paraviridescens
ALKNS91	1393	4	87.0269444	48.7130556	1	T.paraviridescens
ALKNS92	1398	4	87.0277778	48.7135833	1	T.piluliferum
ALFY1	1405	1	89.7280833	46.9803889	1	T.harzianum
ALFY2	1277	1	89.7869444	47.1883056	1	T.asperellum
ALFY3	1183	1	89.8079444	47.2942222	1	T.asperellum
ALFY4	1405	1	89.7280833	46.9803889	1	T.harzianum
ALFY5	1403	1	89.7275278	46.9805278	1	T.harzianum
ALFY6	1277	1	89.7869444	47.1883056	1	T.longibrachiatum
ALFY7	968	1	89.521	45.6664444	1	T.asperellum
ALFY8	968	1	89.521	45.6664444	1	T.citrinoviride
ALFY10	987	1	89.5065	45.5326389	1	T.harzianum
AKTB1	1585	2	87.2103889	48.3576389	1	T.caerulescens
AKTB2	1508	2	87.1106111	48.3200556	1	T.polysporum
AKTB8	1273	3	86.5900278	47.0581389	1	T.paraviridescens
AKTB9	1273	3	86.5900278	47.0581389	1	T.rossicum
BZZY1	2705	2	84.3615556	43.1489722	4	T.polysporum
BZZY2	2482	2	84.1959722	43.0456667	4	T.harzianum
BZZY3	2482	2	84.1959722	43.0456667	4	T.harzianum
BZZY4	2469	2	84.2458889	43.0571944	4	T.harzianum
BZZY5	2705	2	84.3615556	43.1489722	4	T.paraviridescens
CJTC1	1910	2	87.1716944	43.2702778	3	T.harzianum
CJTC2	1922	2	88.1178056	43.8983889	3	T.harzianum
CJTC3	1922	2	88.1178056	43.8983889	3	T.longibrachiatum
CJTC4	1271	2	88.0343611	43.9678611	3	T.paraviridescens
CJTC5	1271	2	88.0343056	43.9678056	3	T.rossicum
CJTC6	1271	2	88.0343611	43.9678611	3	T.rossicum
CJTC30	1924	3	88.1175833	43.8983056	3	T.citrinoviride
CJTC31	1271	3	88.0343056	43.9678056	3	T.harzianum
CJTC32	1925	3	88.1200278	43.8985556	3	T.rossicum
CJTC33	1924	3	88.1175833	43.8983056	3	T.atroviride
CJTC34	1918	3	88.1134444	43.8993611	3	T.paraviridescens
CJTC35	1931	3	88.1161667	43.8996944	3	T.paraviridescens
CJTC36	1918	3	88.1134444	43.8993611	3	T.polysporum

YNTB1	2211	2	84.3467222	43.2051111	2	T.oblongisporum
YNTB2	2453	2	84.3348889	43.1822778	2	T.oblongisporum
YNTB3	2211	2	84.3467222	43.2051111	2	T.oblongisporum
YNTB4	2211	2	84.3467222	43.2051111	2	T.oblongisporum
YNTB5	2211	2	84.3467222	43.2051111	2	T.polysporum
ALKNS93	1393	4	87.0269444	48.7130556	1	T.polysporum
YNTB7	2100	3	84.33075	43.2075833	2	T.oblongisporum
YNTB8	2154	3	84.3353611	43.2058056	2	T.paraviridescens
YNTB9	2039	3	84.3286389	43.2189167	2	T.paraviridescens
YNTB10	2100	3	84.33075	43.2075833	2	T.polysporum
ALKNS94	1374	4	87.0269167	48.7028611	1	T.polysporum
YNTB12	1922	3	84.3109167	43.2128333	2	T.paraviridescens
YNTB13	1872	3	84.3158333	43.2211111	2	T.polysporum
YNTB14	1872	3	84.3158333	43.2211111	2	T.semiorbis
YNTQ1	2330	2	84.3535278	43.65125	2	T.oblongisporum
YNTQ2	2401	3	84.3313333	43.5939722	2	T.citrinoviride
YNTQ3	2481	3	84.3506389	43.5705556	2	T.oblongisporum
YNTQ4	2260	3	84.3821667	43.3731111	2	T.paraviridescens
YNTQ5	1825	3	84.3559444	43.3314167	2	T.asperellum
YNTQ6	2401	3	84.3313333	43.5939722	2	T.citrinoviride
YNTQ7	1825	3	84.3559444	43.3314167	2	T.polysporum
YQRM1	3205	2	84.4264167	43.7480556	2	T.oblongisporum
YQRM2	2517	2	84.4377222	43.6663056	2	T.paraviridescens
YQRM3	3076	2	84.4332778	43.5041111	2	T.polysporum
YQRM4	3076	2	84.4332778	43.5041111	2	T.polysporum
YQRM5	2806	2	84.4674722	43.5104722	2	T.polysporum
YQRM6	2689	2	84.4111944	43.6783889	2	T.polysporum
ALBRK1	791	1	87.0448889	48.1720556	1	T.harzianum
AFKK22	1190	2	89.7564722	47.1114722	1	T.harzianum
AFKK23	1190	2	89.7564722	47.1114722	1	T.harzianum
AFKK24	1260	2	89.7883889	47.1869444	1	T.harzianum
ALKNS26	2001	2	87.2061111	48.4327778	1	T.ghanense
ALKNS95	1368	4	87.0268889	48.7036111	1	T.polysporum
ALKNS96	1368	4	87.0268889	48.7036111	1	T.polysporum
ALKNS97	1363	4	87.0268611	48.7149722	1	T.polysporum
ALKNS98	1363	4	87.0268611	48.7149722	1	T.polysporum
ALKNS99	1393	4	87.0260833	48.7098611	1	T.polysporum
YNTB11	2197	4	84.3446111	43.2034722	2	T.polysporum
ALFY9	1161	1	89.486417	45.2833333	1	T.harzianum
AKTB3	1495	2	87.1098889	48.3203056	1	T.afroharzianum
AKTB5	1797	2	87.1596944	48.4628611	1	T.harzianum
AKTB6	1797	2	87.1596944	48.4628611	1	T.harzianum
AKTB10	1590	2	87.1236111	48.3322222	1	T.viridescens
AKTB11	696	1	86.91475	47.58975	1	T.longibrachiatum
BZHSA1	1009	1	86.0890833	46.6871111	4	T.harzianum

BZHSB1	1227	1	86.348	46.8732222	4	T.ghanense
BZHSB2	1227	1	86.348	46.8732222	4	T.harzianum
WLXG6	1929.4	3	87.3900986	43.3875733	3	T.saturnisporum
CJTC11	2017	2	88.1166667	43.8833333	3	T.afroharzianum
CJTC21	2015	3	88.1166667	43.8833333	3	T.afroharzianum
CJTC22	1972	3	88.1166667	43.8833333	3	T.afroharzianum
CJTC23	1973	3	88.1166667	43.8833333	3	T.afroharzianum
CJTC24	2017	2	88.1166667	43.8833333	3	T.afroharzianum
BZHS2	1570	2	86.5	42.6	4	T.harzianum
YLZSB1	1575	2	81.4666667	43.15	2	T.harzianum
CJTC8	2001	2	88.1166667	43.8833333	3	T.afroharzianum
CJTC19	2001	2	88.1166667	43.8833333	3	T.semiorbis
CJTC29	1972	3	88.1166667	43.8833333	3	T.semiorbis
ALKNS25	1381	4	87.0225278	48.7125556	1	T.citrinoviride
YNTB6	2197	4	84.3446111	43.2034722	2	T.fertile
AKTB4	1492	2	87.1100556	48.3215833	1	T.gamsii
AKTB7	1495	2	87.1098889	48.3203056	1	T.harzianum
BZHSA2	1009	1	86.0890833	46.6871111	4	T.saturnisporum
BZHJ1	2039	2	86.3495278	42.8743056	4	T.harzianum
BZHJ2	2821	2	86.7025278	42.9676667	4	T.koningii
CJTC17	2001	2	88.1166667	43.8833333	3	T.rossicum
CJTC28	1972	3	88.1166667	43.8833333	3	T.rossicum
WLXG1	1928	2	87.39025	43.3985	3	T.harzianum
WLXG2	1928	2	87.39025	43.3985	3	T.harzianum
WLXG3	1982	2	87.3853056	43.3904444	3	T.pleurotum
WLXG4	1968	3	87.3888611	43.3563611	3	T.harzianum
WLXG5	1972	3	87.389	43.3564722	3	T.pleurotum
YLCC1	1430	2	81.1416389	43.5334167	2	T.atroviride
YLCC2	1198	2	81.1526389	43.6103056	2	T.gamsii
YLCC3	1430	2	81.1416389	43.5334167	2	T.harzianum
YLCC4	2117	2	81.1113611	43.47025	2	T.viridescens
YLCC5	2100	2	81.1113333	43.4706389	2	T.viridescens
YLCC6	999	1	81.1621389	43.6453056	2	T.harzianum
YLGL1	867	1	82.5220833	43.4306944	2	T.harzianum
YLGL2	867	1	82.5220833	43.4306944	2	T.harzianum
YLGL3	862	1	82.5221944	43.4309722	2	T.harzianum
YLSH1	2102	2	81.3886944	44.602	2	T.harzianum
YLSH2	2100	2	81.3880833	44.6018889	2	T.harzianum
YLSH3	2109	2	81.3898611	44.5996944	2	T.koningii
YLSH4	2109	2	81.3898611	44.5996944	2	T.longibrachiatum
YLTK1	1625	2	81.8525278	43.3550278	2	T.koningii
YLTK2	1549	2	82.0105556	43.0985833	2	T.rossicum
YLNLT1	1661	2	84.2889444	43.2696111	2	T.hamatum
YLNLT2	1663	2	84.2890556	43.2699444	2	T.viridescens
YLZSA1	1755	2	81.3014444	43.1423056	2	T.atroviride

YLZSA2	2161	3	81.1103611	43.4696944	2	T.viridescens
CJTC12	2034	2	88.1166667	43.8833333	3	T.afroharzianum
CJTC13	2006	2	88.1166667	43.8833333	3	T.harzianum
BZHS3	1570	2	86.5	42.6	4	T.longibrachiatum
CJTC16	2034	2	88.1166667	43.8833333	3	T.longibrachiatum
WLDB2	1736	2	87.2333333	43.4333333	3	T.longibrachiatum
WLDB3	2122	2	87.2666667	43.4	3	T.longibrachiatum
WLXB4	2051	2	87.1666667	43.4166667	3	T.longibrachiatum
WLXB12	2044	2	87.1666667	43.4333333	3	T.longibrachiatum
CJTC26	1972	3	88.1166667	43.8833333	3	T.longibrachiatum
WLDB6	1916	3	87.25	43.4	3	T.afroharzianum
WLXB2	2051	2	87.1666667	43.4166667	3	T.afroharzianum
WLXB5	2065	2	87.1666667	43.4166667	3	T.longibrachiatum
WLXB6	2056	2	87.1666667	43.4166667	3	T.longibrachiatum
WLXB10	2074	2	87.1666667	43.4166667	3	T.longibrachiatum
WLXB13	2047	2	87.1666667	43.4333333	3	T.longibrachiatum
WLDB1	2068	2	87.2666667	43.4	3	T.afroharzianum
WLDB5	2042	3	87.2666667	43.4166667	3	T.afroharzianum
WLDB7	2089	3	87.2666667	43.4	3	T.longibrachiatum
WLXB11	2074	2	87.1666667	43.4166667	3	T.longibrachiatum
YLZSB2	1567	2	81.4666667	43.15	2	T.longibrachiatum
BZHS1	1570	2	86.5	42.6	4	T.asperellum
CJTC10	2016	2	88.1166667	43.8833333	3	T.afroharzianum
CJTC14	2016	2	88.1166667	43.8833333	3	T.longibrachiatum
CJTC20	1972	3	88.1166667	43.8833333	3	T.afroharzianum
CJTC25	2015	3	88.1166667	43.8833333	3	T.asperellum
CJTC27	2015	3	88.1166667	43.8833333	3	T.longibrachiatum
WLDB4	1736	2	87.2333333	43.4333333	3	T.viridescens
WLDB8	2204	3	87.2666667	43.4	3	T.longibrachiatum
WLXB3	2074	2	87.1666667	43.4166667	3	T.afroharzianum
WLXB7	2047	2	87.1666667	43.4333333	3	T.longibrachiatum
WLXB9	2060	2	87.1666667	43.4166667	3	T.longibrachiatum
ALBRC8	775	1	87.0508611	48.1686667	1	T.harzianum
AFKK38	1370	3	89.8761389	47.1955556	1	T.rossicum
CJTC7	1974	2	88.1166667	43.8833333	3	T.afroharzianum
CJTC9	2006	2	88.1166667	43.8833333	3	T.afroharzianum
CJTC15	2016	2	88.1166667	43.8833333	3	T.longibrachiatum
CJTC18	2006	2	88.1166667	43.8833333	3	T.semiorbis
WLDB9	2081	3	87.2666667	43.4	3	T.afroharzianum
WLDB10	2027	3	87.2666667	43.4166667	3	T.afroharzianum
WLDB11	2150	3	87.2666667	43.4	3	T.longibrachiatum
WLXB1	2060	2	87.1666667	43.4166667	3	T.afroharzianum
WLXB8	2070	2	87.1666667	43.4333333	3	T.longibrachiatum
WLXB14	2069	2	87.1666667	43.4333333	3	T.longibrachiatum
WLXG7	1982	2	87.3852778	43.3902778	3	T.pleurotum

**Ecosystem group**	**Word color for the isolates from different collection region**
1. desert steppe	1. Altay
2. emperate steppe	2. Yili
3. coniferous forest	3. Changji and Urumuqi
4. coniferous and Broad-leaved Mixed Forest	4. Bayingolin
